# All types of component malrotation affect the early patient-reported outcome measures after total knee arthroplasty

**DOI:** 10.1186/s43019-019-0006-2

**Published:** 2019-07-05

**Authors:** Mohammad Kamal Abdelnasser, Mohamed Eslam Elsherif, Hatem Bakr, Mohamed Mahran, Moustafa H. M. Othman, Yaser Khalifa

**Affiliations:** 1 Orthopedic Department, Assiut University Hospitals, Assiut, Egypt; 2 Radiology Department, Assiut University Hospitals, Assiut, Egypt

**Keywords:** Knee, Alignment, Arthroplasty, PROMs, Component malrotation

## Abstract

**Purpose:**

Outcomes following total knee arthroplasty (TKA), whether clinical, radiological or survival analysis, have been well-studied. Still, there are some concerns about patient satisfaction with the outcome of the surgery and factors that might contribute to a suboptimal result. This study aims to determine if there is correlation between primary TKA malalignment and early patient-reported outcome measures (PROMs).

**Materials and methods:**

Sixty patients, who had primary TKA and a minimum of 2 years of follow up, were recruited for a detailed clinical and radiological examination. Knee alignment was measured in the coronal, sagittal and axial planes. Normal and the outlier measurements of the patients’ knees were defined and the clinical results (PROMs) compared to see if there was a statistically significant difference.

**Results:**

Correlation between postoperative limb malalignment in the coronal and the sagittal planes and PROMs was not significant. Conversely, there was significant negative correlation between all types of malrotation and PROMs.

**Conclusions:**

Although malalignment has been linked to inferior outcome and implant survival, our results showed that coronal and sagittal limb malalignment has no significant effect on early PROMs. However, all types of component rotational malalignment significantly worsen early PROMs.

## Introduction

Although total knee arthroplasty (TKA) is a well-established orthopedic procedure with documented success; patient dissatisfaction after surgery is still substantially high [1]. Out of each five patients who undergo TKA, one patient is dissatisfied with the results of his or her knee surgery [1]. Outcome after TKA is multifactorial. Patient factors, surgeon factors, implant type, and alignment can influence the outcome after TKA [2].

The real value of patient-reported outcome measures (PROMs) is to have a full view of the patients’ functional limitations, pain levels, mental health state, and expectations from their treatment, thus improving communication between surgeons and patients with the potential for improving postoperative outcomes. Although clinical and radiological evaluations are vital in reaching the proper diagnosis, PROMs should always be assessed as we are treating patients not the x rays [3].

The effect of malalignment on the outcome after TKA remains controversial. The majority of authors link malalignment to decreased implant survival [4, 5]. Others, however, have not noted such correlation [6]. Few researchers [2, 7, 8] have studied the effect of malalignment on PROMs after TKA. Moreover, most of these reports are based on the outcomes at long-term follow up in terms of implant survival, revision rate, and complications.

Outcomes after total knee arthroplasty (TKA), whether clinical, radiological, or survival analysis, have been well-studied. Still, there are some concerns about patient satisfaction with the outcome of the surgery and factors that might contribute to a suboptimal result. This study aims to determine if there is correlation between primary TKA malalignment and early PROMs. Our hypothesis is that malalignment after primary TKA, especially rotational malalignment, has a direct effect on the early PROMs.

## Materials and methods

This is a retrospective cohort study that included 60 patients with primary TKA (60 TKAs). After searching our Arthroplasty Unit database, we contacted 145 patients who had undergone primary TKA and had a minimum follow-up period of 24 months: 60 patients (about 41% of those contacted) were available for clinical and radiological examination. Of these, 46 patients were female (77%) and 14 were male (23%). The mean age was 57 ± 9.6 years. The underlying cause of their knee problems was osteoarthritis (49 patients (82%)) and rheumatoid arthritis (11 patients (18%)). Patients who had undergone revision TKA, previous knee surgery such as high tibial osteotomy, previous knee trauma, or postoperative complications such as infection and intraoperative complications such as fractures were excluded to avoid confounding bias.

The senior author had performed all surgery in these patients using a medial parapatellar approach. Initially the distal femoral cut was made according to the valgus correction angle (VCA), which was individualized according to the difference between the anatomical and mechanical axis as demonstrated on the full-length radiograph obtained in the weight-bearing position. The inter-epicondylar line was used to define the rotation of the femoral component. The tibial cut was made using the external guide. The tibial component was then rotated to the proper rotation depending on the tibial landmark, which is the medial third of the tibial tubercle. A cemented Nex Gen Legacy posterior stabilized (LPS) flex fixed bearing (Zimmer, Warsaw, IN, USA) was used in all knees. According to our institutional postoperative follow-up protocol, all patients were clinically and radiologically evaluated at 6 weeks and at 3, 6, 12, and 24 months.

All 60 patients were recruited for a simultaneous detailed PROMs evaluation and radiological examination including both x-rays and computer tomography (CT) at a minimum of 24 months postoperative follow-up (range 24–132 months postoperative) by an independent researcher in the period from June 2017 to December 2017. Assessments were as follows:

1. Clinical assessment: this was based on the following scores; Knee Society Score (KSS) [9], Knee Osteoarthritis and Disability Outcome Score, Joint replacement (KOOS-Jr) [10, 11], Oxford Knee score (OKS) [12] and Short Form Health Survey (SF-12) [13].
2. Radiological assessment: this was performed as described subsequently.

### Coronal limb alignment

**Fig. 1:**
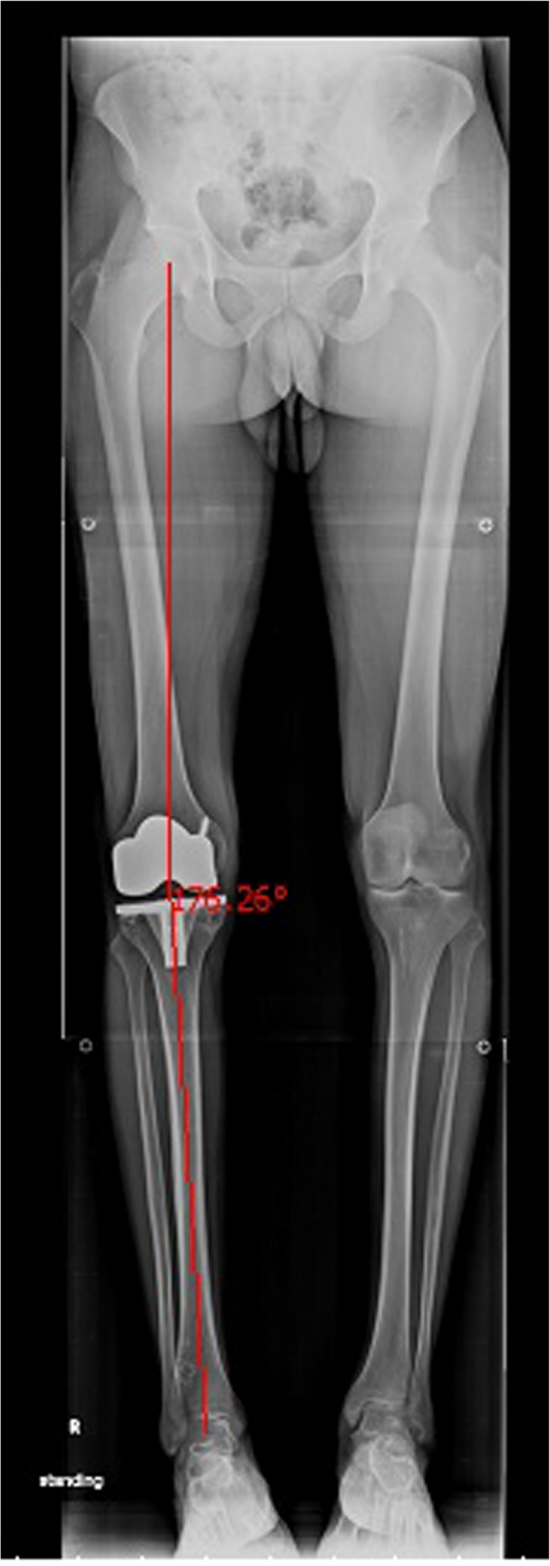
Full-length, standing-position, anterior-posterior radiograph of both lower limbs showing the hip-knee-ankle (HKA) axis

Coronal mechanical alignment of the lower limb was measured by calculating the hip-knee-ankle (HKA) [14, 15] axis angle (Fig. 1), using anterior-posterior-projection, standing position, full-length radiographs of both lower limbs. There is a consensus that postoperative, coronal, limb alignment should be within 0° ± 3° of the mechanical axis [6, 16]. Patients with mechanical axis angles outside this range are considered to have coronal limb malalignment (outliers) [6, 16].

### Sagittal limb alignment

**Fig. 2:**
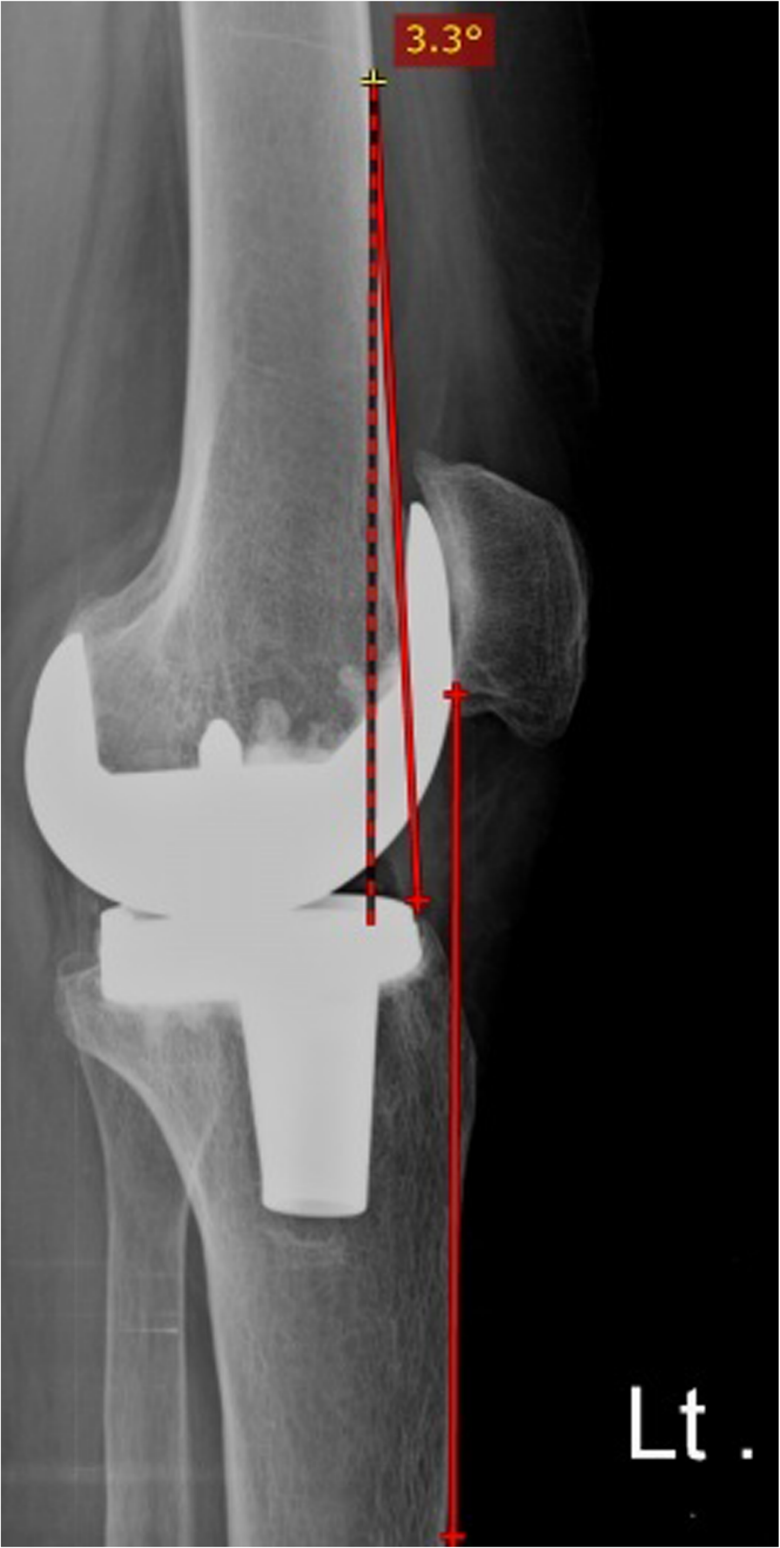
Lateral-projection radiograph of the knee in maximal extension showing the anterior cortical line angle (sagittal limb alignment) by drawing the anterior cortical line of the proximal tibia and of the distal femur and measuring the angle between these two lines

The anterior cortical line angle [17] was calculated to detect the degree of overall sagittal limb malalignment, by marking the anterior cortical line of the proximal tibia and of the distal femur and measuring the angle between these two lines (Fig. 2) on a lateral-projection radiograph of the knee obtained with the knee in maximal extension. Normal sagittal alignment is defined by hyperextension values between 0° and 5°. Flexion values > 0° and hyperextension values > 5° are defined as flexion and hyperextension outliers, respectively [17].

### Component rotational alignment

We followed the protocol described by Berger et al. [18] to evaluate component rotation. The patient was placed supine on the CT scanning table with the affected limb in full extension and adjusted for acquisition of scans perpendicular to the mechanical axis of the knee. A lateral-projection scout film was acquired to ensure that the CT scans were perpendicular to the long axis of the femur and tibia by tilting the CT gantry accordingly. Imaging slices 1.5 mm thick were obtained at four locations: through the epicondylar axis on the femur, though the tibial tubercle, through the top of the tibial plateau, and through the tibial component. Measurements were calculated from the axial slices as described subsequently.

#### Femoral component rotation

**Fig. 3:**
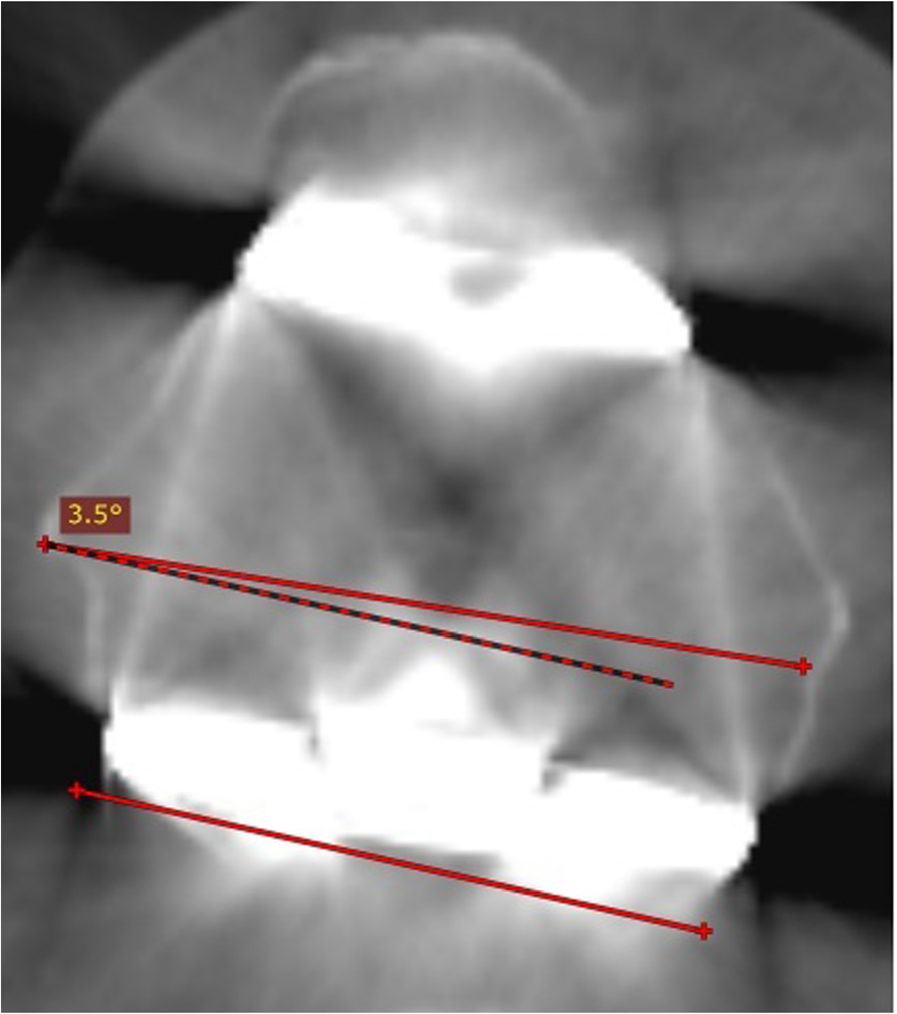
Axial computed tomography slice of a total knee arthroplasty in a male patient, showing neutral femoral component rotation in relation to the surgical transepicondylar axis (sTEA). The sTEA is plotted as a line drawn from the sulcus of the medial epicondyle to the prominence of the lateral epicondyle. The posterior condylar line of the femoral component is plotted as a line drawn from connecting the posterior margins of the medial and lateral posterior component condylar surfaces. The angle between these two lines was then measured and is the posterior condylar angle

Femoral component rotation was assessed using the axial CT image of the femur at the level of the femoral epicondyles. We used the surgical transepicondylar axis, which is the line connecting the medial epicondylar sulcus to the lateral epicondylar prominence. The posterior condylar line of the femoral component was schemed as a line drawn by connecting the posterior margins of the medial and lateral posterior component condylar surfaces. The angle between these two lines was then measured to obtain the posterior condylar angle (Fig. 3). The femoral component was considered neutral when internally rotated 3.5° (±1.2) and 0.3° (±1.2) in relation to the surgical transepicondylar axis (sTEA) in men and women, respectively [18]: patients with a posterior condylar angle outside this range were considered to have femoral component rotational malalignment (outliers) [18, 19].

#### Tibial component rotation

**Fig. 4:**
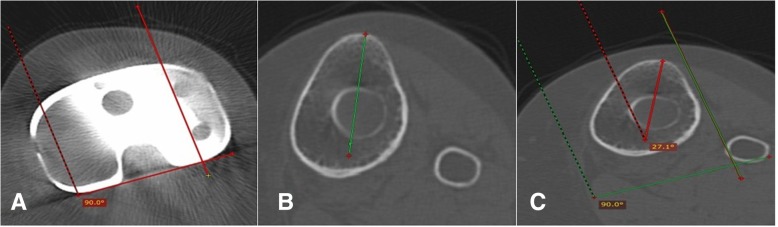
**a** Axial computed tomography (CT) image through the tibial component polyethylene showing the tibial component axis, which is defined as the perpendicular to the posterior margin of the component. **b** Axial CT image through the tibial tuberosity showing the tibial tuberosity axis (T.T.A), which is defined by the line connecting the geometric center of the tibial plateau to the tip of the tibial tubercle. **c** The T.T.A from **b** is superimposed on the tibial component axis (T.C.A) from **a**. The rotation of the tibial component is recorded as the angle subtended by the T.T,A and the T.C.A

Tibial component rotation was assessed from three axial CT images through the tibia. The geometric center of the tibial plateau was located from an axial image just below the tibial component. The geometric center is axially transposed to an axial image through the prominence of the tibial tuberosity and a line is drawn from the geometric center and the tuberosity, the tibial tuberosity axis. The tibial component axis was drawn at the level of the component polyethylene as a line perpendicular to a line drawn along the posterior surface of the component polyethylene. The tibial tuberosity axis was then axially transposed to this image and the angle between these two lines was measured (Fig. 4).

The relationship between the tibial tubercle and tibial articular surface was used to assess tibial component rotation, whether internally or externally rotated. The tip of the tibial tubercle is 18° externally rotated from the tibial articular surface. The tibial component was considered neutral in both men and women when internally rotated 18° (±2.6) in relation to the tip of the tibial tuberosity [18]: patients with an angle this range are considered to have tibial component rotational malalignment (outliers) [18].

#### Combined rotation

Combined component rotation was measured by adding together the femoral and tibial component rotation angles.

#### Rotation mismatch

Component mismatch was calculated by subtracting femoral from tibial component rotation.

##### Statistical analysis

Data were verified, coded, and analyzed using SPSS version 21 (IBM Corp, Armonk NY, USA). Descriptive statistics (mean, standard deviation, median and percentage) were calculated. Statistical significance was tested using the independent *t* test to compare the means of dichotomous variables, and analysis of variance (ANOVA) to compare the means of variables with more than two categories. Correlation was tested to determine the association between variables (Spearman’s rank correlation). A *p* value equal to or less than 0.05 was considered significant.

## Results

Sixty patients were eligible for our study. Limb alignment was assessed in the coronal plane: optimum coronal limb alignment (± 3°) was identified in 39 TKAs (65%), with outliers identified in 21 TKAs (35%) (19 varus TKAs and 2 valgus TKAs). In the sagittal plane, 15 TKAs (25%) were found to have optimum sagittal limb alignment (0–5°) and 45 TKAs were considered outliers. Hyperextension was identified in 31 (51.7%) TKAs and flexion deformity was identified in 14 (23.3%) TKAs.

In the assessment of component alignment in the axial plane, optimum femoral component rotation (3.5° and 0.3° ± 1.2 in relation to sTEA in men and women respectively) was identified in 16 TKAs (26.7%) and 44 TKAs were considered outliers; there were 16 (26.7%) TKAs with an internally rotated and 28 (46.6%) with an externally rotated femoral component. Optimum tibial component rotation (18° ± 2.6 internal rotation in relation to the tibial tuberosity axis in both men and women) was identified in 28 TKAs (46.6%) and 32 TKAs were considered outliers; there were 22 (36.6%) TKAs with an internally rotated and 10 (16.8%) with an externally rotated tibial component. Furthermore, combined component rotation (5 with combined internal rotation and 10 with combined external rotation) was identified in 15 (25%) TKAs and component rotation mismatch was identified in 17 (28.3%) TKAs. Table 1 summarizes normal alignment and outliers in the sagittal, coronal, and axial planes in the cohort studied.

**Table 1:** Results of normal alignment and outliers in the sagittal, coronal, and axial planes in the studied cohort (*n* = 60 patients)

Variable	Category	Number (percentage)	Mean ± SD
Postoperative coronal limb alignment	Normal	39 (65%)	−0.34 ± 0.3
	Varus outliers	19 (30%)	6.74 ± 0.5
	Valgus outliers	2 (5%)	5.83 ± 0.6
Postoperative sagittal limb alignment	Normal	15 (25%)	2.32 ± 0.3
	Hyperextension outliers	31 (51.7%)	14.97 ± 1.9
	Flexion outliers	14 (23.3%)	7.23 ± 1.1
Femoral component rotation	Normal	16 (26.7%)	3.21 ± 0.2
	Internal Rotation outliers	16 (26.7%)	−0.86 ± 0.2
	External Rotation outliers	28 (46.6%)	1.55 ± 0.4
Tibial component rotation	Normal	28 (46.6%)	0.04 ± 0.01
	Internal Rotation outliers	22 (36.6%)	−6.22 ± 0.7
	External Rotation outliers	10 (16.8%)	6.44 ± 1.4
Rotation mismatch		17	9.31 ± 1.9
Combined rotation	Internal Rotation	5	−7.42 ± 1.1
	External Rotation	10	9.43 ± 1.5

Correlation between postoperative limb malalignment in the coronal plane and the sagittal planes and patients’ satisfaction scores was negative and minimal, and was not statistically significant.

**Fig. 5:**
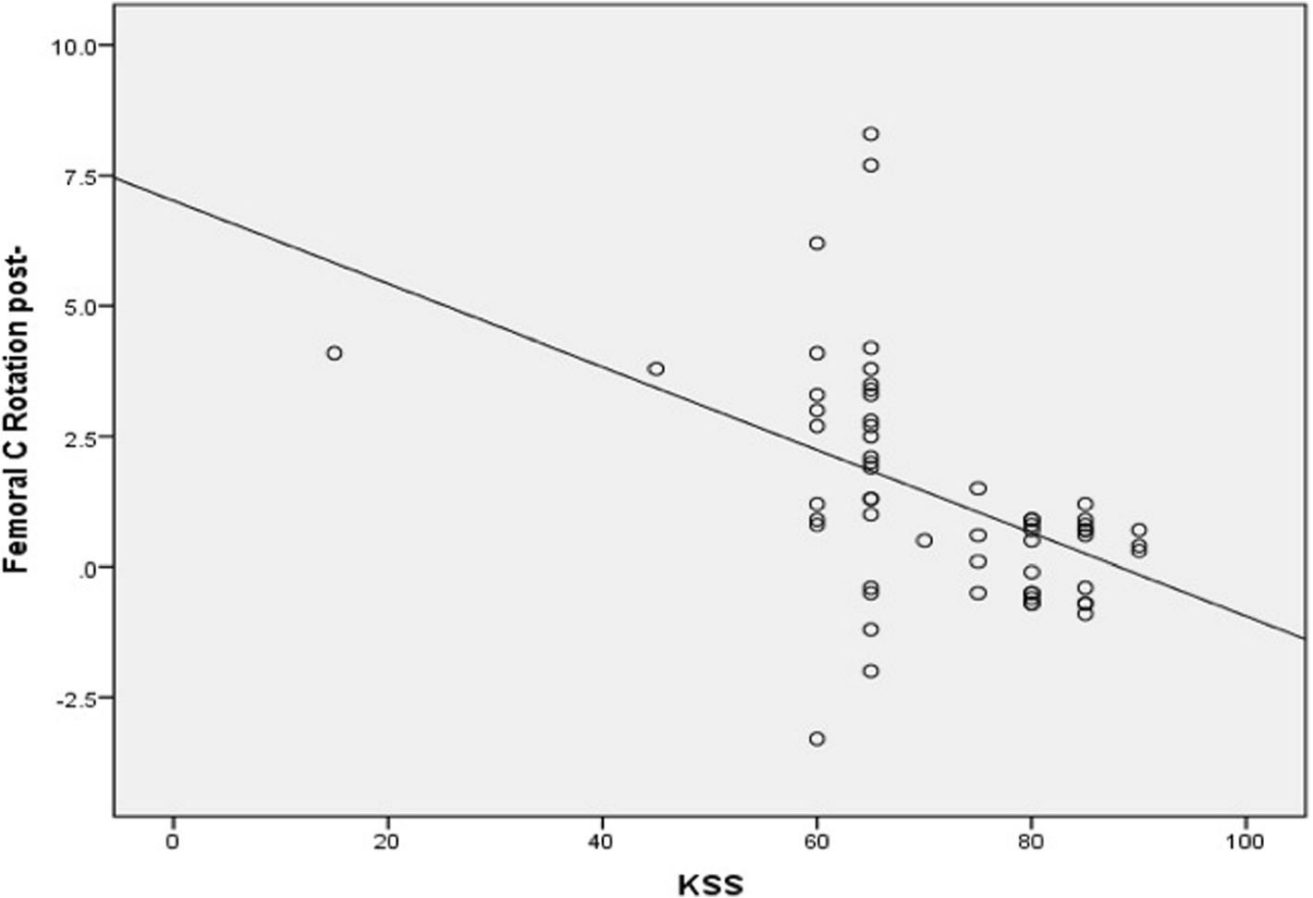
Correlation between femoral component rotation postoperative and the Knee Society Score (KSS)

**Fig. 6:**
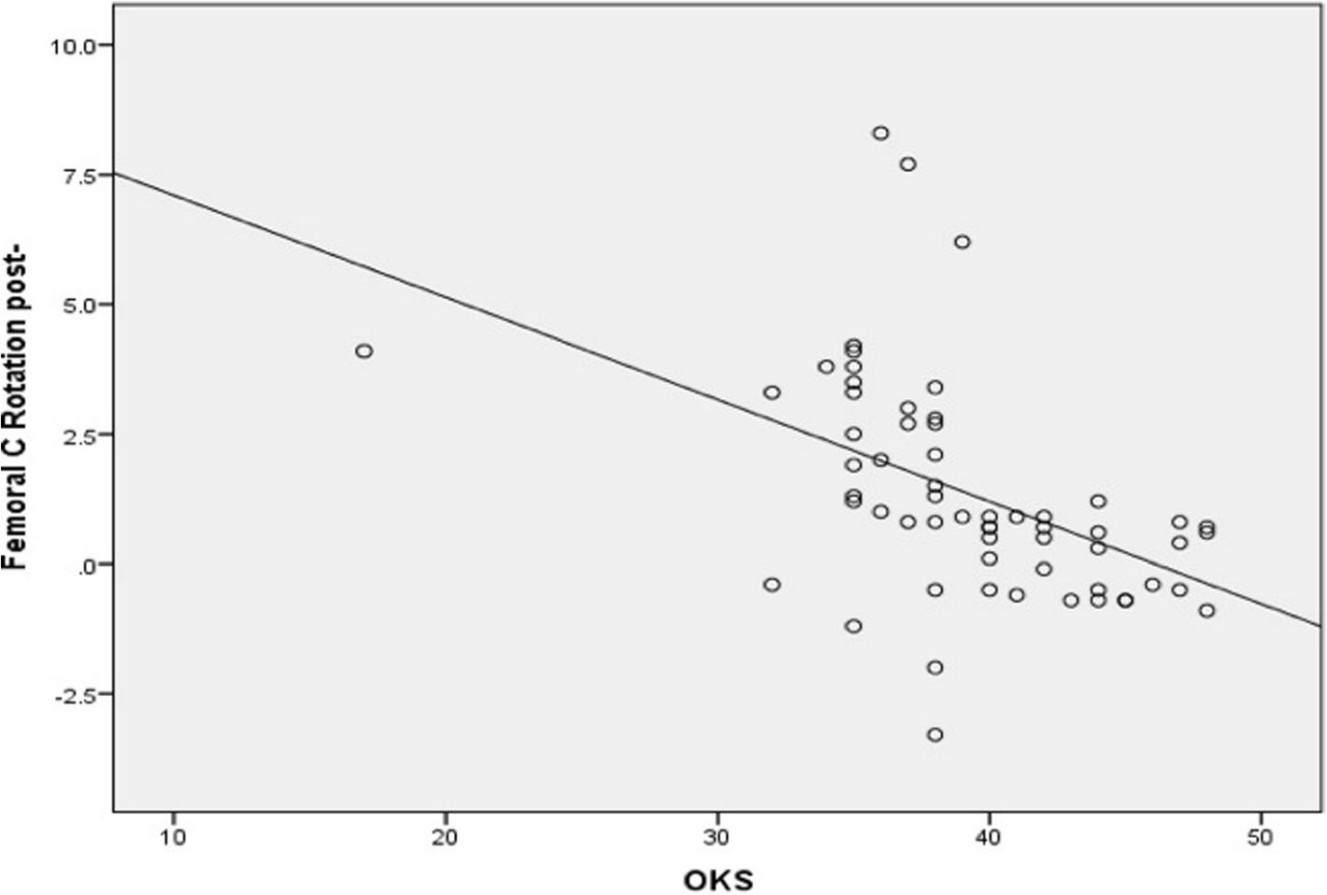
Correlation between femoral component rotation postoperative and the Oxford Knee Score (OKS)

**Fig. 7:**
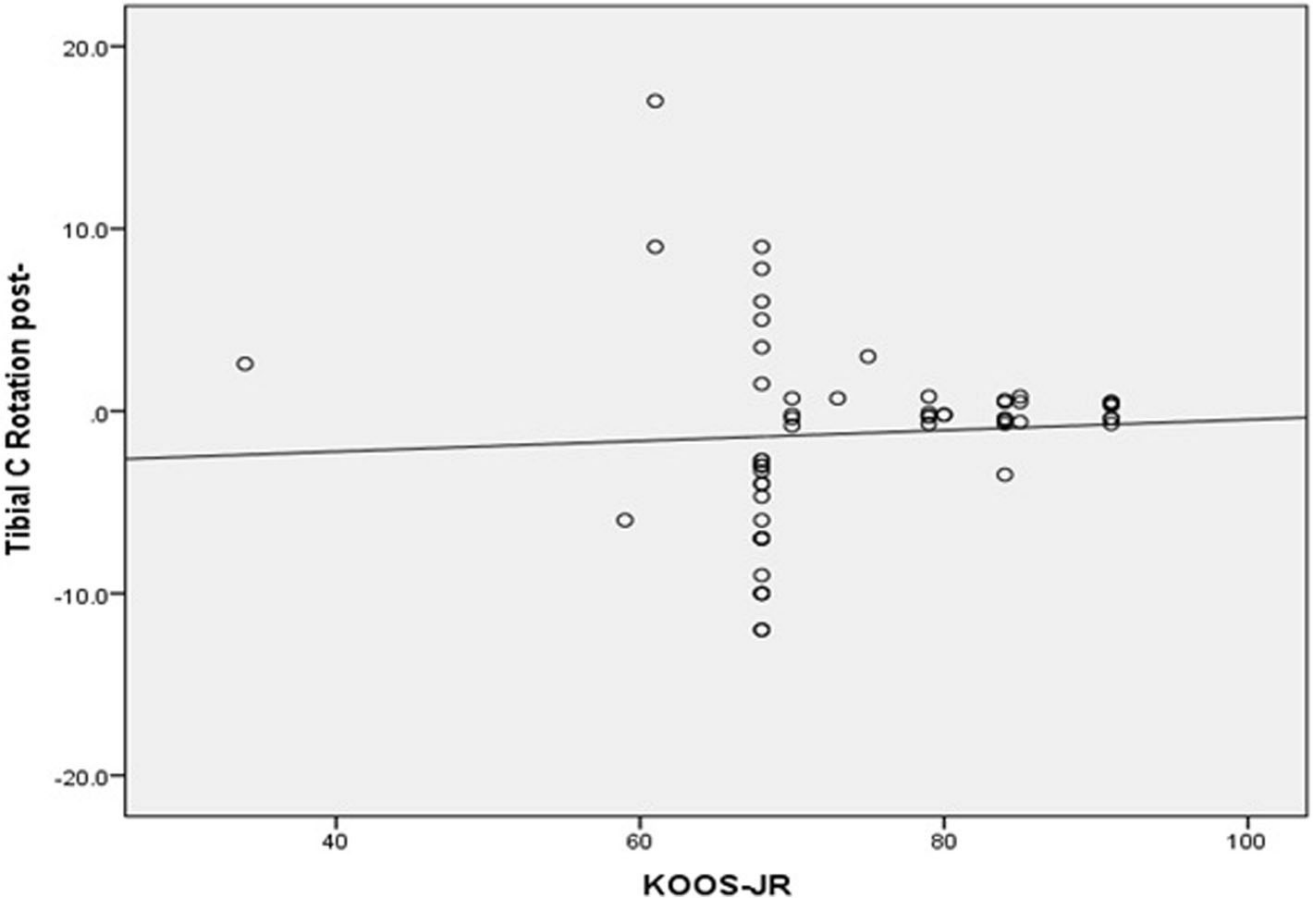
Correlation between Tibial component rotation postoperative and the Knee Disability and Osteoarthritis Outcome Score, Joint replacement (KOOS-JR)

**Fig. 8:**
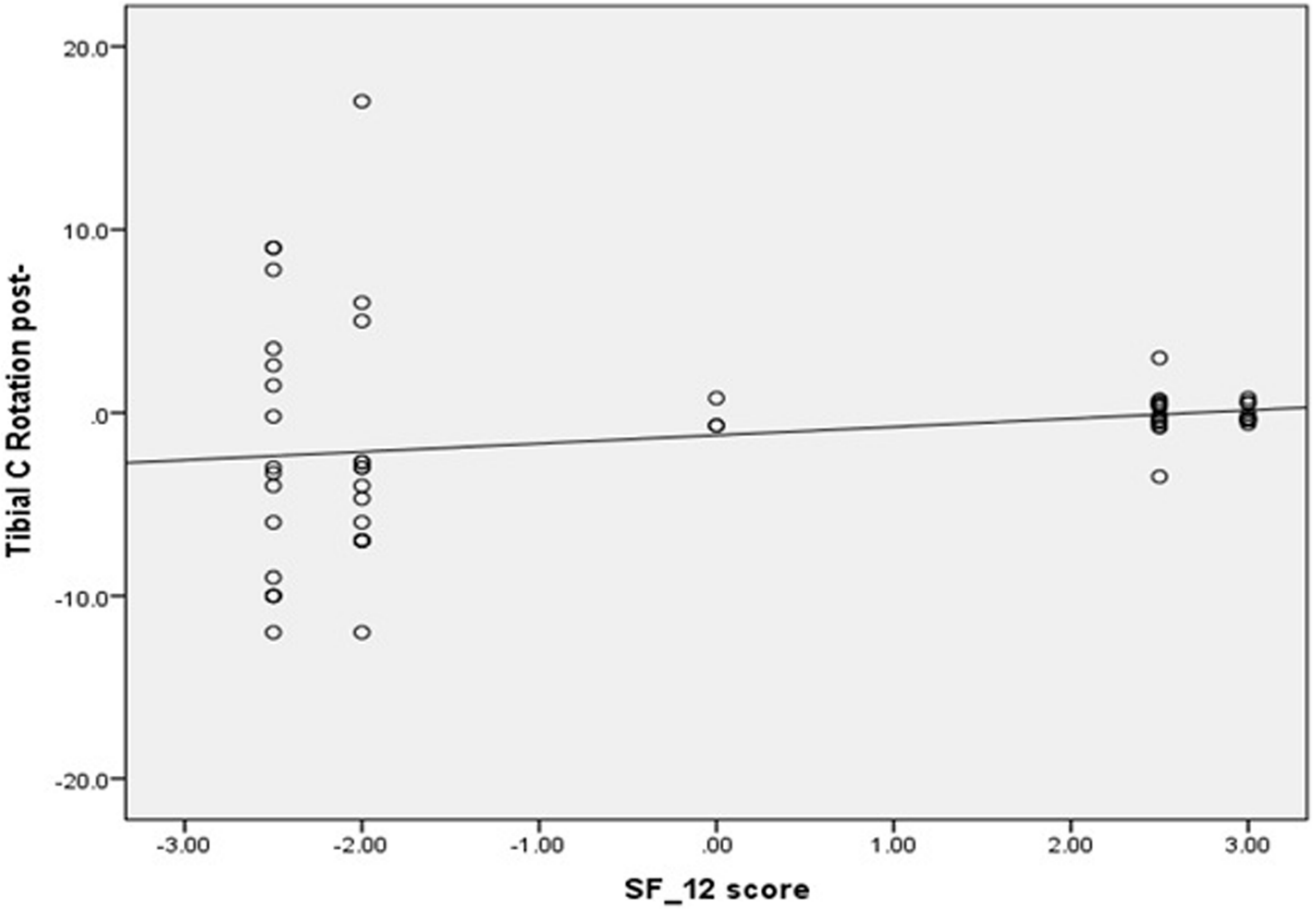
Correlation between Tibial component rotation postoperative and the Short Form Health Survey-12 (SF-12) score

**Fig. 9:**
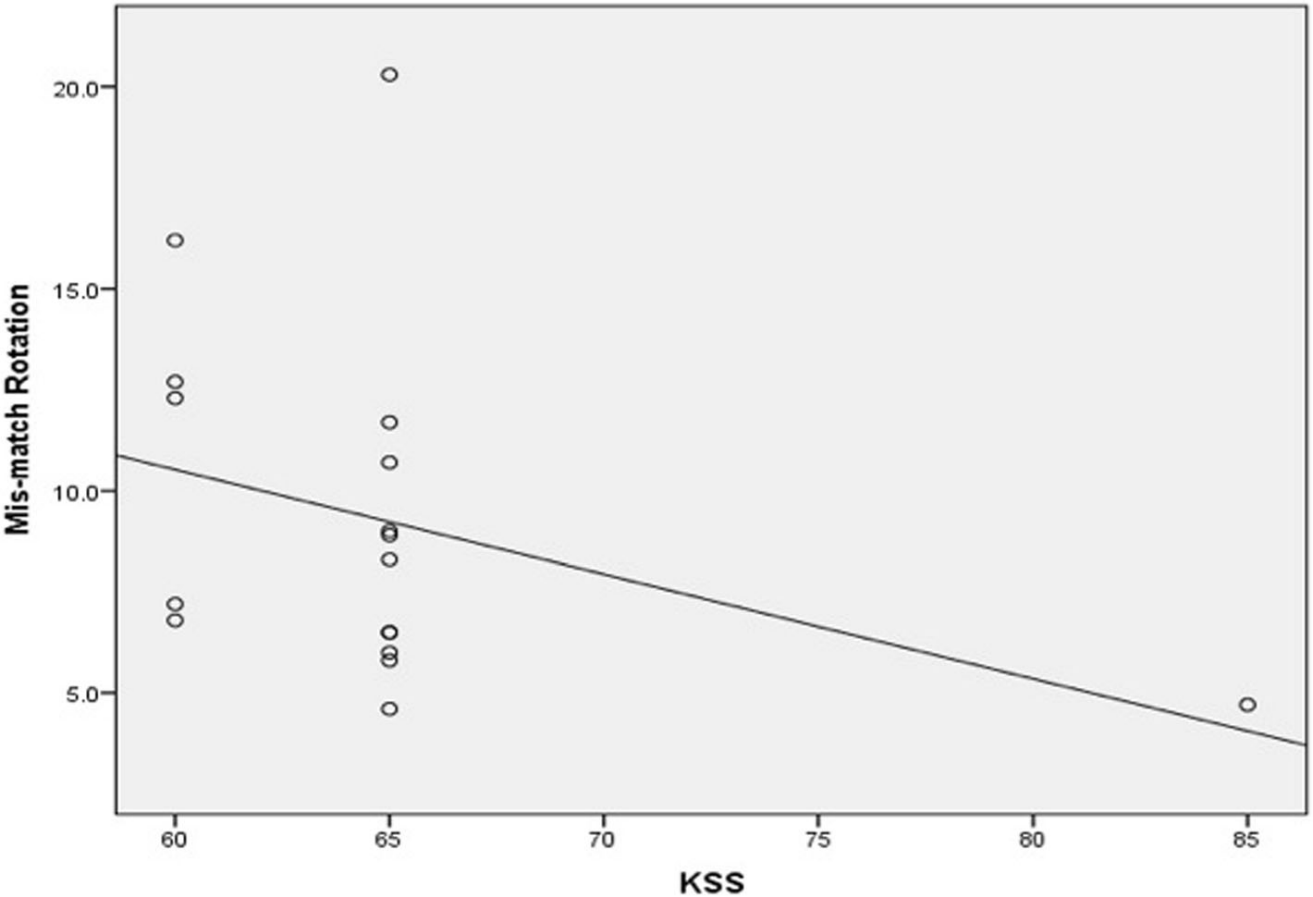
Correlation between degree of rotation mismatch and the Knee Society Score (KSS)

**Fig. 10:**
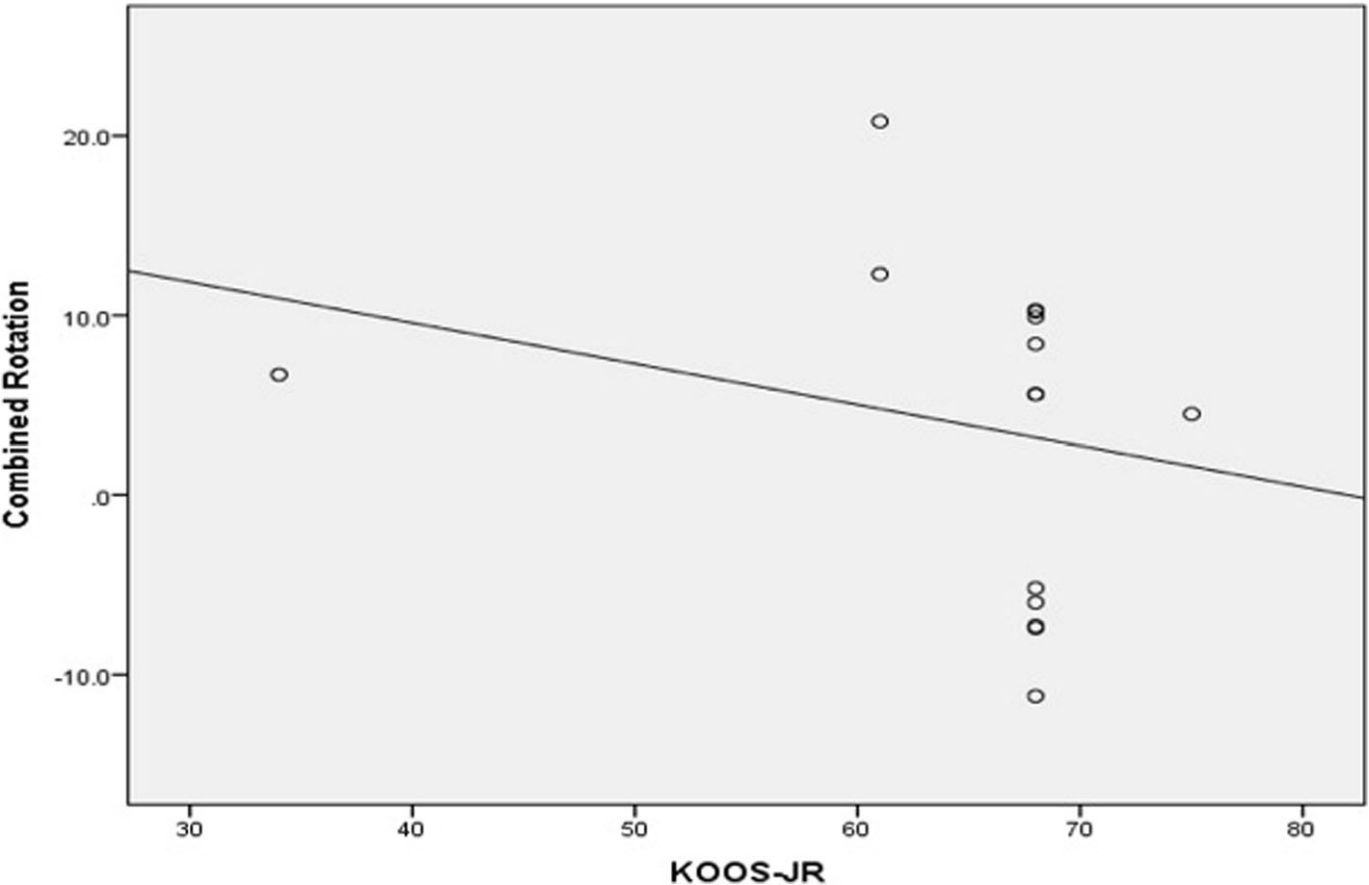
Correlation between degree of combined rotation and the Knee Disability and Osteoarthritis Outcome Score, Joint replacement (KOOS-JR)

Conversely, there was significant moderate negative correlation between femoral component malrotation (internal or external) and all patient satisfaction scores. The correlation coefficient (*r*) was − 0.55 (*p* value <0.0001), − 0.58 (*p* value <0.0001), −0.062 (*p* value <0.0001), − 0.59 (*p* value <0.0001) for KSS, KOOS-JR, OKS, and SF-12 scores, respectively (Figs. 5 and 6). Additionally, there was significant, weak, negative correlation between tibial component malrotation and KOOS-Jr and SF-12 scores. The correlation coefficient (*r*) was − 0.23 (*p* value <0.041) and − 0.33 (*p* value <0.005) for KOOS-JR and SF-12 scores, respectively (Figs. 7 and 8). Also, there was a significant, moderate, negative correlation with component rotation mismatch (Fig. 9). The correlation coefficient (*r*) was − 0.47 (*p* value <0.001) and − 0.49 (*p* value <0.022) for KSS and SF-12 scores, respectively. Furthermore, there was significant, moderate, negative correlation with combined component rotation (Fig. 10). The correlation coefficient (*r*) was − 0.48 (*p* value <0.035) for KOOS-JR (Table 2).

**Table 2:** Correlation between patients’ satisfaction scores and malalignment angle

	KSS	KOOS-Jr	OKS	SF-12
	Correlation coefficient (*r*) and *p* value			
Postoperative coronal limb malalignment	−0.14 (>0.05)	− 0.17 (>0.05)	−0.15 (>0.05)	−0.13 (>0.05)
Postoperative sagittal limb malalignment	− 0.02 (>0.05)	−0.06 (>0.05)	−0.01 (>0.05)	−0.13 (>0.05)
Femoral component malrotation	**− 0.55 (<0.001)**	**−0.58 (<0.001)**	**−0.62 (<0.001)**	**−0.59 (<0.001)**
Tibial component malrotation	0.20 (= 0.059)	**− 0.23 (= 0.041)**	0.18 (= 0.086)	**−0.33 (= 0.005)**
Rotation mismatch	**−0.47 (< 0.001)**	−0.21 (= 0.209)	0.03 (> 0.05)	**−0.49 (= 0.022)**
Combined rotation	0.39 (= 0.077)	**−0.48 (= 0.035)**	0.22 (> 0.05)	0.12 (>0.05)

*KSS* Knee Society Score, *KOOS-Jr* Knee Disability and Osteoarthritis Outcome Score, Joint replacement, *OKS* Oxford Knee Score, *SF-12* Short Form Health Survey-12

*P* value < 0.05 is significant for those data in bold

Mean satisfaction scores for normal alignment and outliers were compared using the *t* test, and there was no significant difference between the two groups in coronal and sagittal alignment. However, there was a significant difference in the case of rotational malalignment of the femur and tibia (Table 3).

**Table 3:** Relationship between malalignment and patients’ satisfaction scores

Variable	Category	KSS score	KOOS-JR score	OKS score	SF-12
Coronal	• Normal	70.90 ± 12.7	74.03 ± 10.7	38.95 ± 5.4	31.64 ± 2.5
	• Outliers	71.67 ± 13.3	74.24 ± 10.3	39.71 ± 4.8	31.14 ± 2.5
	*P* value*	= 0.829	= 0.940	= 0.575	= 0.541
Sagittal	• Normal	71.33 ± 9.9	74.40 ± 9.1	40.20 ± 4.3	31.00 ± 2.5
	• Outliers	71.11 ± 13.7	74.00 ± 10.9	38.89 ± 5.4	31.56 ± 2.6
	*P* value*	= 0.946	= 0.890	= 0.346	= 0.464
Axial Femur	• Normal	78.91 ± 8.5	80.50 ± 8.1	42.19 ± 3.9	33.22 ± 1.8
	• Outliers	62.32 ± 11.2	66.79 ± 7.7	35.82 ± 4.3	29.36 ± 1.3
	*P* value*	**<0.001**	**<0.001**	**<0.001**	**<0.001**
Axial Tibia	• Normal	81.25 ± 5.7	82.29 ± 7.0	43.00 ± 3.2	33.82 ± 0.9
	• Outliers	62.34 ± 10.5	66.94 ± 7.2	35.91 ± 4.1	29.31 ± 1.2
	*P* value*	**<0.001**	**<0.001**	**<0.001**	**<0.001**

*KSS* Knee Society Score, *KOOS-Jr* Knee Disability and Osteoarthritis Outcome Score, Joint replacement, *OKS* Oxford Knee Score, *SF-12* Short Form Health Survey-12

*The *t* test was used to compare the mean difference between the two groups

*P* value < 0.05 is significant for those data in bold

Mean satisfaction scores were significantly different in ANOVA in cases of combined rotation or rotational mismatch compared to cases of normal rotation (Table 4).

**Table 4:** Relationship between normal rotation, combined rotation, mismatch, and patients’ satisfaction scores

Scores (mean ± SD)	Normal rotation (*n* = 28)	Mismatch (*n* = 17)	Combined (*n* = 15)	*P* value*
KSS	81.25 ± 5.7	64.71 ± 5.7	59.67 ± 3.6	**<0.001**
KOOS-JR	82.29 ± 7.1	68.41 ± 4.6	65.27 ± 2.8	**<0.001**
OKS	43.00 ± 3.2	36.88 ± 2.3	34.80 ± 5.4	**<0.001**
SF-12	33.82 ± 0.9	29.29 ± 1.2	29.33 ± 1.3	**<0.001**

*KSS* Knee Society Score, *KOOS-Jr* Knee Disability and Osteoarthritis Outcome Score, Joint replacement, *OKS* Oxford Knee Score, *SF-12* Short Form Health Survey-12

*Analysis of variance was used to compare the mean difference between groups

*P* value < 0.05 is significant for those data in bold

## Discussion

The most important finding of the present study was that we did not identify correlation between limb malalignment in the coronal and sagittal planes and the patient-reported outcome measures, namely KSS, KOOS-Jr, Oxford knee score, and SF-12, after a minimum of 2 years follow up. Conversely, there was positive correlation with all rotational malalignment (femoral malrotation, tibial malrotation, combined rotation, and rotation mismatch) after a minimum of 2 years follow up.

At a minimum of 1 year follow up, Longstaff et al. [7] showed that good alignment can lead to better function, with quicker rehabilitation and earlier hospital discharge. Bach et al. [8], on the other hand, found no correlation between implant alignment and patient quality of life. In a randomized controlled trial, Choong et al. [2] compared alignment between computer-assisted surgery (CAS) and conventional TKA. Neutral mechanical alignment was achieved in CAS more than in conventional TKA. Moreover, there was significant, positive correlation between accurate mechanical alignment after TKA and functional and quality-of-life patient outcomes. At 6 weeks postoperatively, the International Knee Society (IKS) score was significantly better in patients with a mechanical axis within 3° of neutral than when compared to those patients with greater than 3° from neutral, and these results were sustained at all follow-up intervals up until the end of follow up at 12 months. The physical component of the SF-12 scores at the 3-month, 6-month, and 12-month reviews was also significantly better in the group with good alignment.

Blakeney et al. [20] prospectively studied a cohort of 107 patients who underwent TKA. At a mean of 46 months follow up, there was a clinically significant difference in the functional scores in patients treated with CAS versus those treated conventionally. A significant improvement in the OKS was noted when the mechanical axis was within ±3° of neutral, whether navigated or not, versus outliers.

Also, Huang et al. [21] showed that accurate coronal alignment of a total knee prosthesis (to within 3° of neutral) results in better function and better quality of life up to 5 years postoperatively. They compared patients who achieved postoperative alignment within 3° of neutral to those with alignments greater than 3° from neutral, regardless of surgical technique, i.e. CAS versus conventional TKA. The KSS and SF-12 were recorded at 6 weeks, 3 months, 6 months, 12 months, 24 months, and 5 years. Improved pain scores were seen in accurately aligned prostheses up to 2 years postoperatively; however, this did not continue to 5 years.

Contradicting these results, Stucinskas et al. [22] investigated the effect of coronal alignment on muscle strength and function, including 91 TKAs. Range of motion, KSS, and muscle strength measurements were taken at 1 year. They found that moderate varus/valgus malalignment of the mechanical axis, or of individual components, has no relevant clinical effect on function or muscle strength 1 year after TKA. Their rationale is that muscles can adapt to a malaligned axis or components without loss of strength, and can also produce function similar to that in normally aligned TKAs.

Matziolis et al. [23], in a case-control study, compared a group with varus maligned TKA and a control group with well-aligned TKA in patients matched for age, sex, and type of prosthesis. The KSS, Western Ontario and McMaster Universities Osteoarthritis Index (WOMAC), and SF-36 were applied. At a minimum of 5 years follow up, there was no statistically significant correlation between the radiological and clinical data.

Similar to our results, all other reports that studied sagittal component alignment failed to identify correlation between implant malalignemt in the sagittal plane and patient outcome [7, 8].

As regards component rotation, several authors [24–27] have linked component rotational malalignment with inferior clinical and functional outcomes.

Czurda et al. [28] observed a significant association between the risk of postoperative pain and incorrect rotational alignment of the femoral component. In a case-control study, they compared a group of patients with painful TKA and an asymptomatic control group. There was no significant difference between the two groups in mechanical axis, flexion of the femoral component, the dorsal slope, or patella-tracking disorder. However, there was sevenfold elevation in the risk of postoperative pain in those patients with incorrect rotational alignment of the femoral component. Similarly, Barrak et al. [24] found a significant difference between patients with anterior knee pain and an asymptomatic control group in terms of tibial and combined component rotation. Patients with combined component internal rotation have a fivefold increase in the likelihood of experiencing anterior knee pain following TKA compared with those with combined component external rotation. Bell et al. [29] identified excessive malrotation as an important factor in unexplained pain after TKA. On the other hand, excessive external rotation was not found to be a factor in pain following TKR. They identified internal rotation greater than 3.9° femoral internal rotation, 5.8° tibial internal rotation, 8.7° combined component rotation, and 5.8° component mismatch to be excessively rotated. Nicoll et al. [25] found that excessive internal rotation of the tibial component (9°) is a major cause of pain after TKR, whereas external rotation of the tibial component was well-tolerated by most patients. Berger et al. [18], in their series, found that excessive combined internal rotation with angles of more than 7° or 8° resulted in more severe patellofemoral complications, whereas combined external rotation did not.

Also, Lutzner et al. [26] studied 73 patients at an average of 20 months after TKA and found that patients with a postoperative rotational mismatch of more than 10° between the femoral and tibial components showed no improvement and had significantly worse results in the KSS.

Keeping the component rotational mismatch within ±5° during TKA is needed for controlled knee axial rotation during flexion. Mismatch above or below this range results in measurable kinematic differences and causes different patterns of axial rotation motions during passive knee flexion [27].

Although the average amount of internal rotation of the femoral component in our study was 0.86 ± 0.2°, which is far below the average in the aforementioned studies, it was significantly associated with worse functional outcome. On the other hand, the average for internal rotation of the tibial component, combined internal rotation, and mismatch was 6.22 ± 0.7°, 7.42 ± 1.1 °, and 9.31 ± 1.9°, respectively, and these values are close to the average values for malrotation in the same studies.

The results of this study confirm that malalignment in the coronal and sagittal plane has no effect on PROMs at short-term follow up, a finding supported by many authors. It seems that muscles adapt to longstanding deformity in the way that it can easily become accustomed to residual deformity after TKA. Component rotational malalignment alters patellofemoral and knee flexion-extension kinematics from the first day postoperative. This explains the high incidence of early pain and inferior outcome measures when component malrotation is evident.

The main limitation to this study is the short follow-up period. A prospective study design with a longer follow-up period will be needed to track the changes in the PROMs and determine whether or not these changes persist in the midterm and long-term follow up. Another important limitation to this study is that there are many other confounding variables such as body mass index (BMI) and psychological factors, etc., which may affect the outcome after TKA.

## Conclusions

Although malalignment has been linked to inferior outcome and implant survival, our results showed that coronal and sagittal limb malalignment has no significant effect on early PROMs. However, all types of component rotational malalignment significantly worsen early PROMs.

## Data Availability

The datasets obtained and/or analyzed during the current study are available from the corresponding author on reasonable request.

## Abbreviations

ANOVA: Analysis of variance
BMI: Body mass index
CAS: Computer assisted surgery
CT: Computerized tomography
HKA: Hip-knee-ankle axis
IKS: International Knee Society score
KOOS-Jr: The Knee Disability and Osteoarthritis Outcome Score, Joint replacement
KSS: Knee Society Score
OKS: Oxford Knee score
Plain A-P: Anteroposterior x-ray view
PROMs: Patient-reported outcome measures
SF-12: Short Form Health Survey-12
SF-36: Short Form Health Survey-36
sTEA: Surgical transepicondylar axis
TKA: Total knee arthroplasty
VCA: Valgus correction angle
WOMAC: The Western Ontario and McMaster Universities Osteoarthritis Index
